# Editorial: Brain-Computer Interfaces (BCIs) for daily activities: innovations in EEG signal analysis and machine learning approaches

**DOI:** 10.3389/fnhum.2026.1795349

**Published:** 2026-02-20

**Authors:** Baidaa Al-Bander

**Affiliations:** School of Computer Science and Mathematics, Keele University, Keele, United Kingdom

**Keywords:** assistive technology, electroencephalography (EEG), human machine interaction, hybrid brain-computer interfaces (BCIs), interpretable AI, machine learning (ML), neurotechnology and brain-machine interface, real-world BCI applications

Brain-Computer Interfaces (BCIs) are redefining how humans interact with machines by enabling the direct translation of neural activity into meaningful control outputs. By leveraging advances in electroencephalography (EEG) signal analysis, neuroscience, and machine learning, modern BCIs are transitioning from controlled laboratory environments toward practical applications in people's daily lives, including assistive technology, hands-free control, cognitive monitoring, and adaptive human-machine interaction.

This Research Topic was conceived to showcase innovations at the convergence of EEG signal analysis and machine learning methods that push BCIs toward real-world practicality. The collected contributions span a spectrum of advances ranging from algorithmic improvements in EEG decoding accuracy to system-level designs that enhance robustness, usability, and autonomy. Collectively, these works demonstrate how improved signal processing pipelines, predictive modelling strategies, and hybrid system architectures can overcome long-standing challenges such as noise sensitivity, inter-subject variability, and cognitive workload; all critical barriers to everyday BCI adoption.

## Shared autonomy: balancing user intent with adaptive assistance

Human–machine systems designed for daily activities must balance direct user control with autonomous system behaviour to reduce cognitive effort while maintaining responsiveness. Douglas et al. have demonstrated how shared-autonomy frameworks can enable BCIs to operate across multiple levels of user involvement, from direct neural control to high-level goal specification. Rather than relying solely on low-level EEG commands, the proposed approach integrates intent inference with adaptive robotic planning, allowing multiple users to coordinate multiple robots in functional daily tasks. A key insight from this contribution is that increasing autonomy can significantly reduce user workload while preserving task accuracy and efficiency, supporting more sustainable long-term BCI use in real-world settings. This work highlights a broader shift in BCI design toward collaborative control paradigms, where machines proactively assist users instead of acting only as passive command receivers.

## Hybrid interfaces: combining EEG with complementary sensors

Single-modality EEG BCIs are often limited by noise, signal ambiguity, and performance variability across users and environments. Hybrid approaches that fuse EEG with additional sensing modalities offer a promising solution, as demonstrated by Coutray and their team. Coutray et al. have proven how integrating EEG with eye-tracking improves command disambiguation, interaction speed, and overall control reliability, particularly in immersive virtual reality environments. This hybrid design reduces reliance on high-precision EEG decoding alone and enables more natural, intuitive hands-free interaction, expanding BCI applicability beyond clinical contexts into entertainment, accessibility, and smart-environment control. This contribution reinforces the growing consensus that multimodal BCIs are more scalable, resilient, and user-friendly than EEG-only systems.

## Machine learning for EEG-based diagnosis and real-world signal decoding

Machine learning, particularly deep learning, plays a critical role in extracting meaningful patterns from noisy EEG signals. Alarfaj et al. have demonstrated how neural network architectures can improve classification accuracy for clinically relevant EEG patterns, including automated seizure detection. Beyond clinical value, these results highlight an important cross-domain insight: techniques developed for EEG-based diagnosis can strengthen BCI decoding pipelines, enabling more reliable real-time intent recognition. This convergence suggests that BCI research and EEG-driven health monitoring can mutually reinforce each other, accelerating progress toward adaptive, health-aware neural interfaces.

## Understanding cognitive dynamics for more adaptive BCIs

Robust BCI performance depends not only on decoding algorithms but also on the user's cognitive and neural state. Mohamed et al. have provided evidence that baseline neural oscillations, particularly pre-cue alpha activity, influence event-related desynchronisation (ERD) strength, a core signal used in motor-imagery BCIs. A concrete implication of this finding is that BCIs could dynamically adjust classification thresholds, training protocols, or feedback timing based on real-time cognitive state estimates, potentially improving accuracy, consistency, and user learning rates. These insights support the development of state-aware BCIs that adapt decoding strategies in response to moment-to-moment brain dynamics, improving reliability in naturalistic, everyday environments.

## Interpretable prediction of performance under physiological stress

Beyond EEG decoding alone, combining physiological stress markers and subjective workload measures enables more comprehensive prediction of user performance in demanding tasks. Wei et al. have demonstrated that interpretable machine-learning models can forecast task outcomes while revealing which physiological factors most strongly influence performance. This is particularly relevant for real-world BCI deployment, where fatigue, stress, and cognitive overload can degrade system reliability. A key insight is that interpretable predictive models can support adaptive intervention strategies, such as adjusting task difficulty or providing rest prompts, while maintaining transparency and user trust.

## Emerging themes and future directions

Across the contributions, several overarching themes emerge:

- Multimodal integration: combining EEG with complementary sensors enhances decoding robustness and interaction flexibility.
- Adaptive autonomy: systems that balance user intent with automated assistance reduce workload and improve task efficiency.
- Machine-learning innovation: deep and interpretable models improve accuracy while supporting transparency and trust.
- Cognitive context awareness: accounting for neural and psychological state enables more reliable and personalised BCI performance.

Together, these themes suggest a transition from static, single-user BCIs toward adaptive, context-aware, and multi-agent systems designed for long-term everyday use.

## Conclusions

The contributions in this Research Topic provide a snapshot of the evolving landscape of EEG-based BCIs, highlighting a shift toward systems that are adaptive, interpretable, multimodal, and resilient in real-world environments. By advancing both core signal-decoding methodologies and application-focused system designs, these studies collectively help bridge the gap between experimental neuroscience and practical daily-life BCI deployment. As the field continues to mature, deeper integration across machine learning, neural engineering, human-computer interaction, and applied clinical research will be essential for ensuring that BCIs become not only technically powerful but also accessible, trustworthy, and impactful for diverse user populations.

